# Fish-Waste-Derived Gelatin and Carbon Dots for Biobased UV-Blocking Films

**DOI:** 10.1021/acsami.2c11749

**Published:** 2022-07-25

**Authors:** Carlotta Campalani, Valerio Causin, Maurizio Selva, Alvise Perosa

**Affiliations:** † Department of Molecular Sciences and Nanosystems, Università Ca’ Foscari di Venezia, Via Torino 155, 30172 Venezia Mestre, Italy; ‡ Dipartimento di Scienze Chimiche, Università di Padova, via Marzolo 1, 35131 Padova, Italy

**Keywords:** biobased films, waste, gelatin, carbon dots, UV-shield, UV-blocking

## Abstract

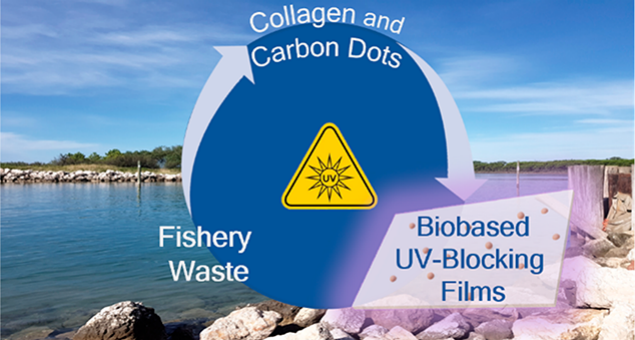

The fish industry produces every year huge amounts of waste that represent an underutilized source of chemical richness. In this contribution, type I collagen was extracted from the scales of *Mugil cephalus* and carbon dots (CDs) were synthesized from the scales of *Dicentrarchus labrax.* These materials were combined to make hybrid films with UV-blocking ability, by casting a mixture of gelatin, glycerol (15%), and CDs (0, 1, 3, and 5%). The films were fully characterized from the mechanical, morphological, and optical point of view. Here, 40 μm thick films were obtained, characterized by a high water solubility (70%); moreover, the presence of CDs improved the film mechanical properties, in particular increasing the tensile strength (TS) up to 17 MPa and elongation at break (EAB) up to 40%. The CDs also modulated water vapor permeability and the thermal stability of the films. From the optical point of view, with just 5% loading of CDs the films blocked almost 70% of the UV radiation with negligible change in transparency (88.6% for the nonloaded vs 84.4% for 5% CDs) and opacity (1.32 for nonloaded vs 1.61 for 5% CDs). These types of hybrid biobased films hold promise for the production of sustainable UV-shields both for human health and for prolonging the shelf life of food.

## 1 Introduction

Interest in preventing overexposure to ultraviolet (UV) light is growing. Blocking UV light, in fact, protects human skin, eyes, immune and biological systems,^1^ packaged food and pharmaceuticals, artifacts from fading, etc. UV radiation is commonly divided into three main types: UV-A (400–315 nm), UV–B (315–280 nm), and UV–C (280–100 nm). These radiations can cause significant damage to the eyes and the skin, leading to possible premature aging and skin cancer thanks to their ability to penetrate the human dermis.^2^ In addition, protection from UV radiation allows to extend the lifetime of many products such as drugs and foods.^3^ All these reasons have led to an increasing demand for transparent UV-shielding materials to protect both humans and sensitive substances. Currently two types of UV protecting materials have been produced: using either inorganic or organic photoactive compounds. A plethora of different inorganic nanomaterials (such as ZnO^4^,^5^ CuO,^6^ CeO_2_,^7^ and TiO_2_^8^) have been described for this purpose. However, these inorganic oxides tend to aggregate if the nanoparticle loading exceeds moderate threshold concentrations affecting the transparency in the visible region. To overcome this problem, organic materials are preferable due to their more favorable optical properties^9^.^10^ Unfortunately, organic dyes are often toxic and can degrade during their exposure to light.^11^ In the past few years, many researchers have aimed at obtaining materials with UV-blocking properties without compromising the visible light transparency while contextually reducing toxicity^12^^13^.^14^ Our ongoing studies on the valorization of fish waste with a view toward the circular economy, along with the need to develop transparent UV-shielding materials with high environmental- and bio-compatibility inspired us to develop functional films from fish-waste derived photoactive carbon dots (CDs) embedded in a fish gelatin matrix. Up to date, in fact, there are some instances of UV-blocking CD-based films, including a few made from biobased materials, but never in an integrated biowaste-to-product approach such as in the present case.

In this work, we therefore explored the valorization of fishery waste for the production of gelatin/CDs films that act as barrier for UV radiation. Gelatin was extracted from mullet (*Mugil Cephalus*) scales and CDs were synthesized using bass (*Dicentrarchus labrax*) scales as the carbon source.

Using discarded fish scales to produce high added-value materials addresses the need to recover waste and convert it into new materials, chemicals, and products toward a more circular economy. In this context, the fishery industry can provide dozens of million tons per year^15^ of biowaste that represent a virtually inexhaustible source of sustainable chemical richness.^16^ Nowadays, such waste is usually processed mainly for the production of low-tech fishmeal and fertilizers. While the composition of fish residues can vary according to species, sex, age, time of the year, and geographic area, nonetheless several valuable molecules and compounds are contained in all fish biowaste. In particular, fish waste can be used as a natural source for oils, collagen, chitin, pigments, and gelatin.^15^

Gelatin is a biodegradable protein derived from the partial hydrolysis of collagen that is gaining increasing interest in a large variety of fields such as photography, pharma, and cosmetics. This is due to its favorable properties such as high water-solubility, thermo-reversible sol–gel transition, nontoxicity, high mechanical strength, and elasticity in the dry state.^17^ Traditionally gelatin is produced from collagen derived from bovines and swine and the annual world output is around 326 000 tons. However, mammalian gelatin has some problems mainly due to the transmission of bovine spongiform encephalopathy disease (BSE), commonly known as mad cow disease, as well as to religious and social issues. For these reasons, fish gelatin is gaining prominence in recent years, especially when derived from the byproducts of the fish processing industries.^18^ The waste derived from the fish production and processing is, in fact, a problem of growing significance and this abundance may pose an environmental hazard.^19^ The use of this type of waste for the synthesis of renewable products, such as biopolymers, could therefore represent a dual opportunity. The abundance of fish byproducts such as scales, skin, and bones can be, in fact, a great and sustainable source of gelatin. In recent years, several researchers focused on the preparation and characterization of fish gelatin. The majority of the studies performed gelatin extraction from skin and bones of different fish species^18,20,21^ while the production from scales was reported using black tilapia,^22^ bass and mullet,^23^*Labeo rohita*,^17^ sea bream,^24^ deep-sea redfish,^25^ and some others.

Gelatin has been extensively studied for its film-forming ability and applicability for protecting foods^26^ but also as carrier of bioactive compounds,^27^ suggesting the possibility to use it as an alternative to synthetic plastics.

CDs are a new class of carbon nanoparticles with excellent photostability, low costs, low toxicity, and high sustainability.^28,29^ The outstanding properties of these nanoparticles are raising considerable interest in a wide range of applications, from biomedical^30,31^ to energy-related fields.^32^ Among these, biobased luminescent CDs are promising for UV-shielding. These carbon nanomaterials were exploited for the preparation of UV-blocking films using them as additives in poly(vinyl alcohol)^2,33,34,35^ nanocellulose^32,36^ starch,^37^ or carboxymethylcellulose.^38^ In one of our previous papers, we demonstrated the possibility to obtain luminescent CDs from bass scales with high in photoelectron transfer properties.^39^ This new class of CDs has been fully characterized from the morphological and optical point of view, highlighting a natural nitrogen doping without the need of external doping agents.

In this framework, the absence of completely renewable UV barriers made using fish-derived gelatin and carbon dots clearly emerges, especially from waste sources.

Therefore, the purpose of this work was to demonstrate that the abundance and chemical richness of fishery waste (namely mullet and bass scales) can be exploited to produce high-tech and high added-value materials and go a step toward the concept of the Circular Economy. In particular, gelatin was obtained from mullet scales with a three steps chemical extraction, and it was used to produce bioderived films with UV-shielding properties. The UV-blocking ability of the material was achieved adding carbon dots as additives in the film-forming solution. The carbon nanoparticles were also synthesized starting from fishery waste (bass scales) using a hydrothermal treatment.

## 2 Materials and Methods

### Materials

All the reagents, of analytical grade and used without further purification, were purchased from Merck Life Science S.r.l. (Milano, Italy). Milli-Q water (obtained with a Merck Millipore C79625 system) was used as a solvent throughout the experiment.

The fish scales were from sea bass and mullet and were sourced from a local market. Prior to use, the scales were thoroughly washed with water and dried overnight in a vacuum oven at 70 °C before storage at −18 °C.

### CDs Synthesis

Bass-scale CDs were synthesized according to our previously reported work.^39^ In summary, a Teflon-lined autoclave was charged with 2 g of dried and ground sea bass scales and 20 mL of Milli-Q water. After heating at 200 °C for 24 h, the obtained suspension was filtered. Residual water was removed by rotary evaporation and CDs were obtained as a brown solid (30–50% yield).

### Fish Gelatin Extraction

Gelatin was extracted from mullet scales by adapting the method described by Niu et al.^40^ The scales were first rinsed and dried, then immersed in aqueous NaOH 0.3 M (1:6 w/v) for 1.5 h at room temperature for the removal of noncollagenous proteins. The scales were then filtered and rinsed until neutral pH. Next, the biomass was soaked in aqueous HCl 0.2 M (1:6 w/v) for 1.5 h at room temperature for the removal of minerals and then filtered and rinsed to neutral pH. The scales were then immersed in acidic Milli-Q water (pH = 5 with HCl, 1:4 w/v) at 78 °C for 1 h to extract collagen, the solid was filtered off, and the resulting liquid was centrifuged at 6000 rpm for 30 min to remove impurities. The solution was then cast in plexiglass molds and water was allowed to evaporate at room temperature for 20 h. Gelatin gave a transparent solid film in 14% yield.

### Fish Gelatin Characterization

#### Bradford Protein Assay

The Bradford method^41^ was used to determine the protein content in the extracted gelatin. The protein standard used to obtain the calibration curve was bovine serum albumin. The mixture of gelatin solution (2.5, 4, 5.5, 7, and 9 μg/mL) and Coomassie Blue dye (200 μL) was incubated for 30 min before the absorbance at 595 nm was recorded with a UV–vis spectrophotometer (Shimadzu UV-1800). Two different batches of mullet scales were used for gelatin extraction, and the assay was run in duplicate.

#### Molecular Weight Distributions by Gel Electrophoresis

Gelatin solutions were prepared at two different concentrations (1 and 2 mg/mL) by dissolving gelatin into Milli-Q water (60 °C for 10 min) and adding dithiothreitol (350 mM final concentration). SDS-PAGE (sodium dodecyl sulfate-polyacrylamide gel electrophoresis) was conducted using as the standard a molecular weight marker with 5–245 kDa (Sharpmass VI-protein MW marker). The samples were denaturated at 90 °C for 10 min and the loading volume was 10 μL. Stacking gel and running gel used were 4% and 6% respectively and the instrument was set at 20 mA current. Following the separation, the separating gel was stained with Coomassie Blue dye (2.5 μL) to identify the bands. After the process, the electrophoresis gel was stained in a methanol solution to remove residual buffer and dye. The percentage proportion of each band was estimated using Imagej software. All samples were analyzed in duplicate.

#### Gel Permeation Chromatography

Gel permeation chromatography (GPC) was performed on an Agilent Infinity 1260 system equipped with refractive index detector and using an injection volume of 20 μL and a flow rate of 1 mL/min. A Phenomenex PolySep linear was used as column maintaining a constant temperature of 40 °C during the analysis. An aqueous solution of LiCl 0.1 M was used as eluent and polyethylene glycol was used as standard. The sample was prepared dissolving 2 mg/mL of mullet scales collagen directly in the eluent solution at 50 °C.

#### Viscoelastic Properties

Gelatin was dissolved in Milli-Q water (60 °C for 10 min) to yield a 6.67% (w/v) gelatin solution. Viscoelastic studies were carried out on a Rheometer Kinexus lab+ (Malvern Instruments) by using a parallel plate with a diameter of 2 cm, a gap of 0.2 mm and a constant strain of 5 Pa. Analyses were performed by heating the solution in two ways: from 5 to 40 °C at a scan rate of 5 °C/min and a frequency sweep of 1 Hz and from 12 to 90 °C at a scan rate of 5 °C/min and a frequency sweep of 0.3 Hz. The elastic modulus (*G*′, Pa), viscosity modulus (*G*″, Pa), and angle phase (δ = *G*′/*G*″, deg) were calculated and plotted as a function of the temperature.

#### Gelatin–CDs Film Formation

The gelatin–CDs films were prepared by the casting technique. The film-forming solution was obtained dissolving in Milli-Q water 2% (w/v) of fish gelatin at 45 °C for 30 min under continuous stirring. Glycerol was added as plasticizer in 15% (w/w_gelatin_) and CDs as additive at different percentages (1, 3 and 5% w/w_gelatin_). Then, aliquots of 8 mL of film-forming solution were poured in Plexiglas molds (7 × 5 cm) and dried at room temperature (25 °C) for 20 h.

### Gelatin–CDs Film Characterization

#### Film Thickness and Mechanical Properties

Film thickness was measured using a hand-held micrometer (TESA, sensitivity of ±0.01 mm) averaging nine different points.

Tensile strength (TS, MPa), elongation at break (EAB, %), and Young modulus (YM) were determined using an INSTRON3345 instrument following ASTM standard method D882-97. Samples were cut into strips of 15 × 50 mm, which were fixed on the grips of the device with an initial grip distance of 30 mm and a crosshead speed of 1.0 mm/min until the films were broken. The samples were not conditioned before the measurements, which were, however, performed all in a single session, at the same temperature and relative humidity. Five replicates were acquired for each sample. Reproducibility of the measurements was checked preparing a new lot of 5% CD containing film and of neat gelatin. Results confirmed those obtained on the pristine lots. Relative errors were 20% for tensile strength, 8% for the Young modulus, and 14% for elongation at break.

#### Water Solubility

The method reported by Gómez-Estaca et al.^21^ was applied with some modifications to determine the water solubility (WS%) of the films. Four cm^2^ portions of the films were dried in a vacuum oven (20 mbar) at 70 °C for 24 h (constant weight was achieved). The samples were then weighted, placed in beakers with 15 mL of Milli-Q water, which was sealed, and stirred at 25 °C for 15h. The solution was then filtered to recover the undissolved film that was then desiccated in a vacuum oven (20 mbar) at 70 °C for 24 h. Water solubility was then calculated using eq 1, where *W*_0_ referred to the initial weight of the film (as dry matter) and *W*_f_ was the undissolved desiccated film residue weight. All tests were carried out in triplicate.
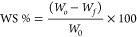
1

#### Water Vapor Permeability (WVP)

WVP values were determined according to ASTM method E96 [ASTM E96-95] using 5 mL cups. Every cup containing anhydrous CaCl_2_ (RH% = 0%), was covered using a portion of film sealed using silicone vacuum grease and was placed inside a desiccator that contained a saturated solution of NaCl (RH = 75%) at 25 °C. Cups were weighted every hour for the first 7 h and finally after 24h. The slope of the weight increase per hour (g/h) divided by the exposed film area (m^2^) yielded the water vapor transmission rate (WVTR).^42^ WVP was then calculated using eq 2 where WVTR is the water vapor transmission rate, *t* is the thickness of the films (m), *P* is the saturation vapor pressure at 25 °C (Pa), *R*_1_ is the RH in the desiccator (0.75), and *R*_2_ is the RH in the cup (0). The difference between desiccator RH and anhydrous calcium chloride corresponds to water vapor partial pressure, 1753.53 Pa and is the driving force of water vapor transition.^43^ All tests were carried out in duplicate.
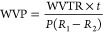
2

#### Optical Properties (UV–Visible, Color)

UV–visible spectra of the films were recorded on a Shimadzu UV-1800 spectrophotometer both in absorption and transmittance mode at wavelength from 800 to 190 nm. All tests were carried out in triplicate.

Opacity was then evaluated using eq 3, where *A*_600_ is the absorbance value at 600 nm wavelength and *t* is the film thickness (mm).
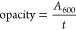
3

Color measurements were performed using a spectrophotometer Konica-Minolta Co Ltd. (Osaka, Japan), model 2600d with an illuminant D65 and 10 degrees observer. All data were extracted using the instrument software. Parameters such as L* (lightness), a* (redness/greeness) and b* (yellowness/blueness) were used to express the results. A white plate was used as standard (*L*_std_* = 99.27, *a*_std_* = −0.07 and *b*_std_* = −0.06). The total color difference (Δ*E*), yellow index (YI), and white index (WI) were calculated using eq 4, 5 and 6.
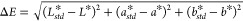
4
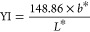
5
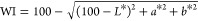
6

#### Differential Scanning Calorimetry (DSC) and Thermogravimetric Analysis (TGA)

The thermal properties and stability of film samples were determined by differential scanning calorimetry and thermogravimetry. For DSC measurements, a TA Instruments 2920 apparatus was used. The film samples (4–5 mg) were weighted into aluminum pans and accurately sealed, then scanned over the range −20 to 200 °C at heating rate of 10 °C/min. An empty aluminum pan was used as reference. *T*_g_ was measured with the graphical construction shown in Figure S3 of the Supporting Information.^44^ TGA measurements were carried with a TA Instruments 2960 apparatus out in a temperature range from 20 to 800 °C with a heating rate of 5 °C/min under a nitrogen flow of 1 mL/min. In order to quantify the repeatability of the measurements, three replicates were recorded for two of the samples (1% and 5% CD). The standard deviation of *T*_g_ was ±4 °C. TGA curves for replicate measurements were superimposable, so an instrumental uncertainty of ±1% for weight loss and ±0.5 °C were used.

#### Transmission Electron Microscopy (TEM) and Scanning Electron Microscopy (SEM)

Transmission electron microscopy (TEM) observations of the CDs and fish gelatin/CDs composites were conducted at 120 kV. For the TEM images of the films, small droplets of the film forming solutions were deposited on TEM grids and dried at room temperature for 24 h to form ultrathin films transparent to the electron beam. Dimensions of nanoparticles and aggregates were estimated using Imagej software.

Morphology of the surface of film samples were visualized using a scanning electron microscope (SEM-FEG Zeiss instrument) operating at 10000 kV and at different magnifications. Samples were cut into small pieces and placed on stub with double-sided carbon tape.

#### Statistical Tests

Two sample *t* tests, with pooled variance, using a 2-tailed distribution were applied at a 95% confidence level and were used to evaluate the statistical significance of comparisons between the data regarding different samples.

## 3 Results and Discussion

Gelatin was extracted from mullet scales using a three-step chemical protocol (deproteinization, demineralization and hydrothermal extraction) in 14% yield. Carbon dots (CDs), used as additives for the subsequent preparation of the films, were obtained as 10 nm nanoparticles using a hydrothermal treatment in autoclave exploiting bass scales as the carbon source as reported in one of our previous papers.^39^

The film forming solution was prepared by mixing together the fish gelatin, glycerol (15%) and CDs in different loading percentages (0, 1, 3 and 5%); casting of this mixture yielded the films.

### Mullet Scales Gelatin. Bradford Protein Assay, and Molecular Weight Distribution by Gel Electrophoresis

The effective protein content in the gelatin extracted from mullet scales as measured by the Bradford protein assay was equal to 52%, indicative of the presence of residual water bound to the protein network and/or the presence of impurities, possibly minerals or chitin. As will be shown later, however, TGA showed that both the pristine gelatin and the CDs-containing samples had a similar content of water and of inorganic impurities; therefore, no composition effect was expected to jeopardize reproducibility.

**Figure 1:**
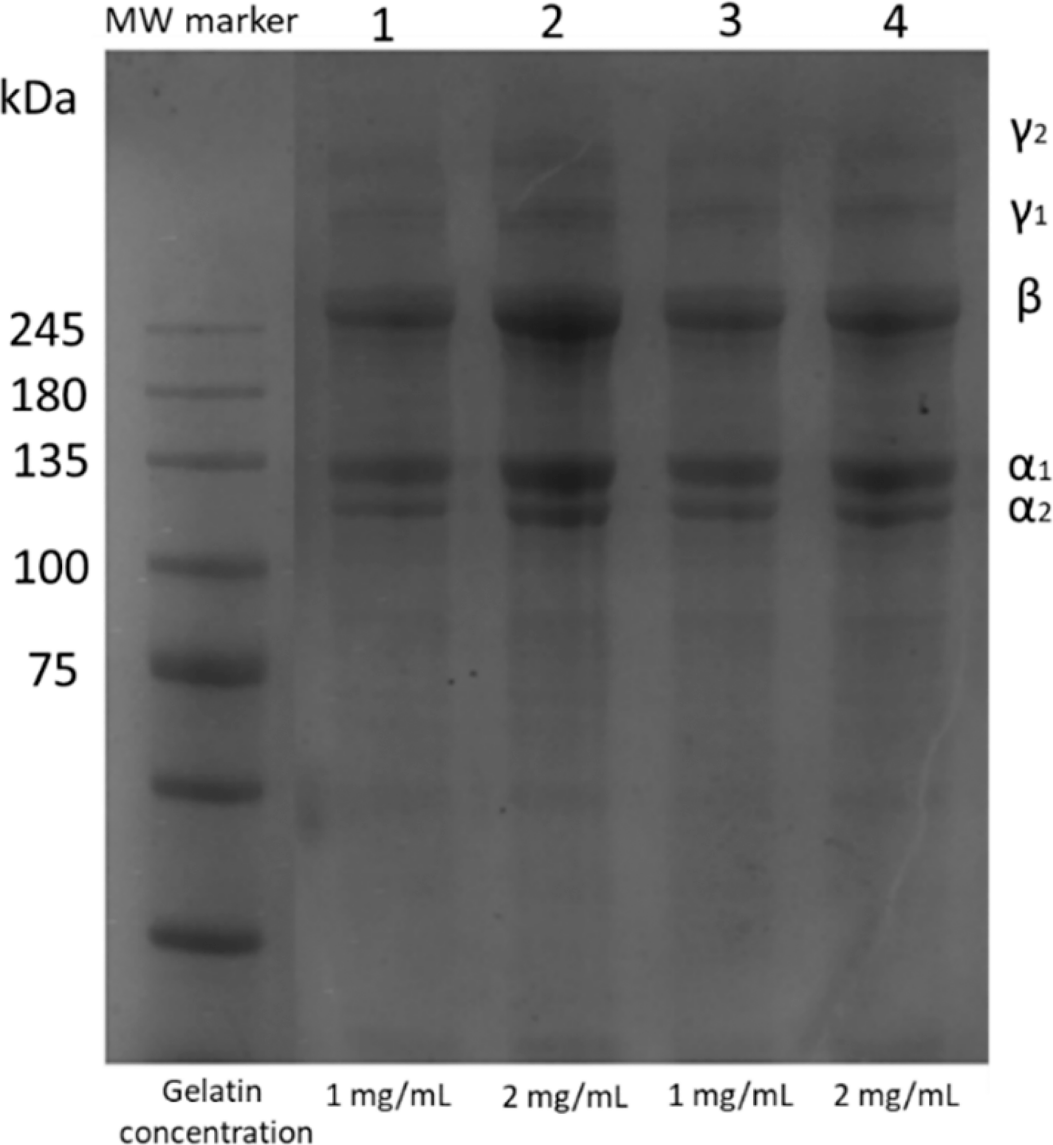
Gel electrophoresis of the gelatin extracted from mullet scales.

Gel electrophoresis was consistent with a typical pattern of type I collagen. Four bands correspondent to α_1_, α_2_, β, and γ chains were identified in each sample (Figure 1). In particular, the α_1_ chain is visible at 135 kDa, the α_2_ at 118 kDa, the β at 245 kDa, and the γ bands at higher molecular weights. The less intense bands at lower molecular weights (around 70 kDa) were attributed to the hydrolysis of collagen in smaller fragments.

The average contribution of α_1_, α_2_, β, and γ (total γ_1_ + γ_2_) chains further confirmed the type I nature of the extracted collagen. In fact, as in type I collagen, the content of the α_1_ chains is usually twice the α_2_.

### Gel Permeation Chromatography

To further confirm the molecular weight distribution of the extracted collagen, gel permeation chromatography (GPC) was conducted. With this technique only a broad peak was observed, probably comprehensive of all the molecular weights highlighted from the electrophoretic analysis. This data is an additional proof that the latter is a more suitable technique for the evaluation of collagen’s MWs. However, the information obtained via GPC was consistent and complementary to electrophoresis: the GPC peak was centered at 124 kDa, showing a MW close to the ones found for the α chains (135 and 118 kDa) meaning that the most abundant form of collagen in the sample was the α chain.

### Viscoelastic Properties

The viscoelastic properties of gelatin were analyzed by determining the elastic modulus (*G*′), the viscous modulus (*G*″) during heating of an aqueous gelatin solution. The measurement was performed at 2 different frequencies, viz. 0.3 and 1 Hz, obtaining similar results. Initially, the profile showed values of *G*′ > *G*″ indicating an elastic and solid-like behavior which was maintained up to ca. 26 °C. At this temperature a crossover point (*G*′ = *G*″ and δ = 45°) was identified corresponding to the gel-point of the solution, after which the gelatin showed a liquid-like behavior. In Figures S1 and S2, the rheological profiles of aqueous gelatin samples are reported. The observed crossover point could be ascribable to the denaturation temperature (*T*_d_) of collagen and this result is similar to those reported for collagen of carp scales.^45^*T*_d_ of collagen from marine fish scales is usually about 26–29 °C,^46^ thus being generally less thermally stable than mammalian one (*T*_d_ ≈ 41 °C).^47^ This behavior can be due to the low imino acid content (hydroxyproline and proline) of marine fish collagen:^48^ heat resistance, in fact, is known to increase with the imino acid content. As reported from Cao et al.^23^ and Thuy et al.^46^ the imino acids content for gray mullet scale gelatin is around 171–197/1000 residues. The low *T*_d_ of mullet scales collagen highlights the possibility to extract gelatin at lower temperature compared to mammalian one, giving an economic advantage for the use of fish scale as a raw material.

### Film Thickness and Mechanical Properties

Table 1 shows the mechanical properties and the thickness of the gelatin films with different percentages of CDs. The control film (fish gelatin + 15% glycerol) was rather ductile (elongation at break = 27.5%) and exhibited a tensile strength = 12.5 MPa in accordance with fish gelatin films produced by Nur Hanani et al.^49^ The addition of small amounts of CDs (1–3%) produced a slight increase in the tensile strength (*t* test *p* value = 0.039) of the material, with rather constant elongation at break and Young modulus. However, the addition of 5% of CDs produced an evident plasticizing effect, as can be seen from the significant decreases in both tensile strength (*p* < 0.003 in a *t* test comparing 1 or 3%CD-containing films with 5%CD-containing sample) and Young’s modulus (*p* value <0.00001), together with a noticeable increase in elongation at break (*p* value = 0.0262 in a comparison with the 1% CD-containing sample). Therefore, CDs seem to have a double role. When particles are small, they have a reinforcing effect, analogous to that of other nanofillers. When agglomeration of the nanoparticles in the matrix becomes significant, such as in the case of 5% CD-containing materials (see the section Transmission Electron Microscopy (TEM) and Scanning Electron Microscopy (SEM) for TEM micrographs), they no longer stiffen the structure, but they instead act as plasticizers.

**Table 1:** Thickness, Tensile Strength (TS), Elongation at Break (EAB), and Young Modulus (YM) of Gelatin Films at Different CDs %, with Values Given as Mean ± Standard Deviation

% CDs	thickness (μm)	TS (MPa)	EAB %	YM (MPa)
0	40 ± 3	12 ± 2	27 ± 4	160 ± 13
1	41 ± 4	17 ± 3	32 ± 4	171 ± 14
3	41 ± 4	17 ± 3	28 ± 4	185 ± 15
5	42 ± 2	10 ± 2	40 ± 6	80 ± 6

### Water Solubility and Water Vapor Permeability

Water solubility (WS) and water vapor permeability (WVP) are important measures of water resistance and integrity of a film. The control film without CDs exhibited a normally high WS of 70.06% but still lower than the one reported for gelatin films made from rohu (91.49%),^50^ cod (88%),^51^ squid (>90%),^52^ catfish (83.3%),^53^ and cuttlefish (96.02%).^54^

The formation of low molecular weight monomers and small peptides during film formation is probably the main reason for the high water-solubility. These low molecular weight components immobilized in the film, account for the water-soluble protein component of the film.^55^ Despite the water-soluble nature of CDs due to the abundance of polar groups on their surface, the solubility of gelatin films was reduced by adding small amounts (1% and 3%) of carbonaceous nanoparticles. This trend was ascribed to the cross-linking effect of the hydroxyl groups present on the surface of the CDs^39^ that initiate the formation of a network which incorporates low molecular fractions leading to the decreased water solubility of gelatin.^49,56^ On the other hand, adding higher concentrations of nanoparticles (5%, entry 4 in Table 2) the WS returned to the value of the control film (ca. 70%), probably because the hydrophilic nature of CDs prevailed making the films more water-soluble. Another argument is that the crystal structure of fish gelatin protein can be disrupted by the nanoparticles, resulting in increased water solubility of the film.^49^

**Table 2:** Water Solubility (WS%) and Water Vapor Permeability (WVP) of the Composite Gelatin/CDs Films at Different CDs %, with Values Given as Mean ± Standard Deviation

entry	CDs %_w/w_	WS %	WVP 10 ^–7^ (g h^–1^ m^–1^ Pa^–1^)
1	0	70.1 ± 0.2	1.05 ± 0.05
2	1	54.9 ± 0.4	0.776 ± 0.006
3	3	60.0 ± 0.3	0.75 ± 0.01
4	5	69.9 ± 0.3	0.75 ± 0.01

As shown in Table 2, WVP decreased when CDs were added to the gelatin meaning that the films behaved as a stronger barrier against water vapor. Also this behavior can be explained by considering that CDs can cause a decrease in the diffusion rate of water molecules through the films, resulting in lower WVP values^57^ by their ability to enhance the cross-linking of gelatin, and as a consequence, to decrease the free volume of the polymeric matrix. Nanoparticles, in fact, can lead to a long and tortuous transport path of water vapor in thin films, which is one of the main reasons for the reduction of WVP.^58^

### Optical Properties

**Figure 2:**
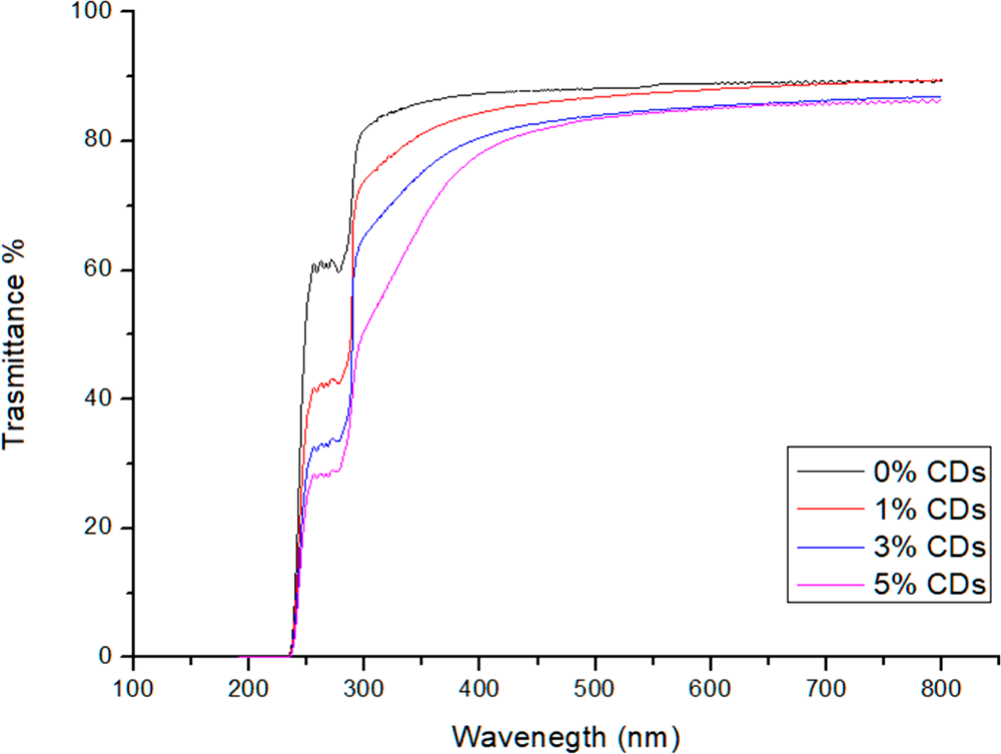
UV–visible transmittance spectrum of gelatin films with different concentrations of CDs (0% black line, 1% red line, 3% blue line, and 5% pink line).

A crucial insight into both structure and optical properties of the films was obtained by UV–vis spectroscopy. In Figure 2, the spectra in transmittance of the films with different percentage of CDs are shown. The thickness of the analyzed samples was ∼40 μm. The dependence of the film’s optical transparency in the visible region (light transmittance at 550 nm^33^) against different CDs content is reported in Table 3. The loading of CDs affects the visible transparency of the film limitedly (*p* value = 0.004): it decreases from 89% (nonloaded film) to 84% (5% of CDs). The decrease in the transparency of the material is probably related to the agglomeration of the nanoparticles inside the gelatin matrix (see Transmission Electron Microscopy (TEM) and Scanning Electron Microscopy (SEM) for TEM micrographs).

**Table 3:** Colorimetric Data and Shielding Behavior (Transmittance % at 275, 300, 365, and 550 nm) of Fish Gelatin–CDs Films at Different % w/w of CDs^a^

								transmittance (%)			
CDs %	L*	a*	b*	Δ*E*	YI	WI	opacity	275 nm	300 nm	365 nm	550 nm
0	98.56	–0.12	0.49	0.89	0.74	98.47	1.3	60.6	81.9	86.4	88.6
1	96.77	–0.58	6.47	7.01	9.95	92.74	1.4	42.8	73.9	82.1	87.5
3	88.75	0.04	25.4	27.55	42.60	72.22	1.5	33.6	65.3	76.7	84.8
5	88.86	–0.07	30.34	32.12	50.80	67.68	1.6	28.9	50.7	70.6	84.4

a The results were expressed as L* (lightness), a* (redness/greeness), and b*(yellowness/blueness). The total color difference (Δ*E*), yellow index (YI), and white index (WI) were calculated using eqs 4, 5, and 6.

The addition of the carbon nanoparticles marginally increases (*p* value = 0.0005) the opacity of the films, causing a maximum increase from 1.3 (0% CDs) to 1.6 (5% CDs).

Concerning the UV-blocking ability of the gelatin–CDs films it can be easily seen from the transmittance UV–vis spectra that the addition of the carbon nanoparticles caused an increase in their shielding properties. Three different wavelengths were chosen to represent the three portions of the UV spectrum: 365 nm for UV-A, 300 nm for UV-B, and 275 nm for UV-C. The transmittance percentage values at these wavelengths are reported in Table 3 versus the CDs content. These data highlight how higher percentages of CDs lead to a higher UV-shielding behavior, reaching the ability to block almost 70% of the UV light.

Colorimetric parameters were assessed to obtain essential information regarding the optical properties of the films. In Table 3, the colorimetric data are reported. L* values (lightness), that vary from 0 (black) to 100 (white), were >88% for all the measured samples. Increasing the percentage of CDs, indeed, the b* values increased, indicating a predominance of more yellow. This is clearly highlighted also from the growing yellow index values and the consequent decrease of the whiteness index (Δ*E*, YI, and WI calculated with eqs 4, 5, and 6).

### Differential Scanning Calorimetry (DSC) and Thermogravimetric Analysis (TGA)

Differential scanning calorimetry (DSC) was used to determine the thermal properties of the gelatin–CDs films and the corresponding glass transition temperature (*T*_g_). The *T*_g_ is defined as the temperature at which the polymer relaxes and changes from the glassy state to the elastic one, for a given heating rate due to the onset of long-range coordinated molecular motion of the amorphous structure. The measurement of *T*_g_ was complicated by the onset of the wide endothermal peak due to water evaporation. However, an estimation could still be made. The fish gelatin film without the addition of CDs resulted to have a *T*_g_ of about 25 °C, in accord with the transition from solid-like to liquid-like behavior observed by rheology. Upon addition of the CDs, an increase in the *T*_g_ value was observed. In fact, the glass transition temperature increased to about 50 °C, independent of CDs content (Figure S4). This trend evinced the ability of CDs to form additional intermolecular forces inside the gelatin matrix, as reflected also by the changes in the mechanical properties of the materials.

**Figure 3:**
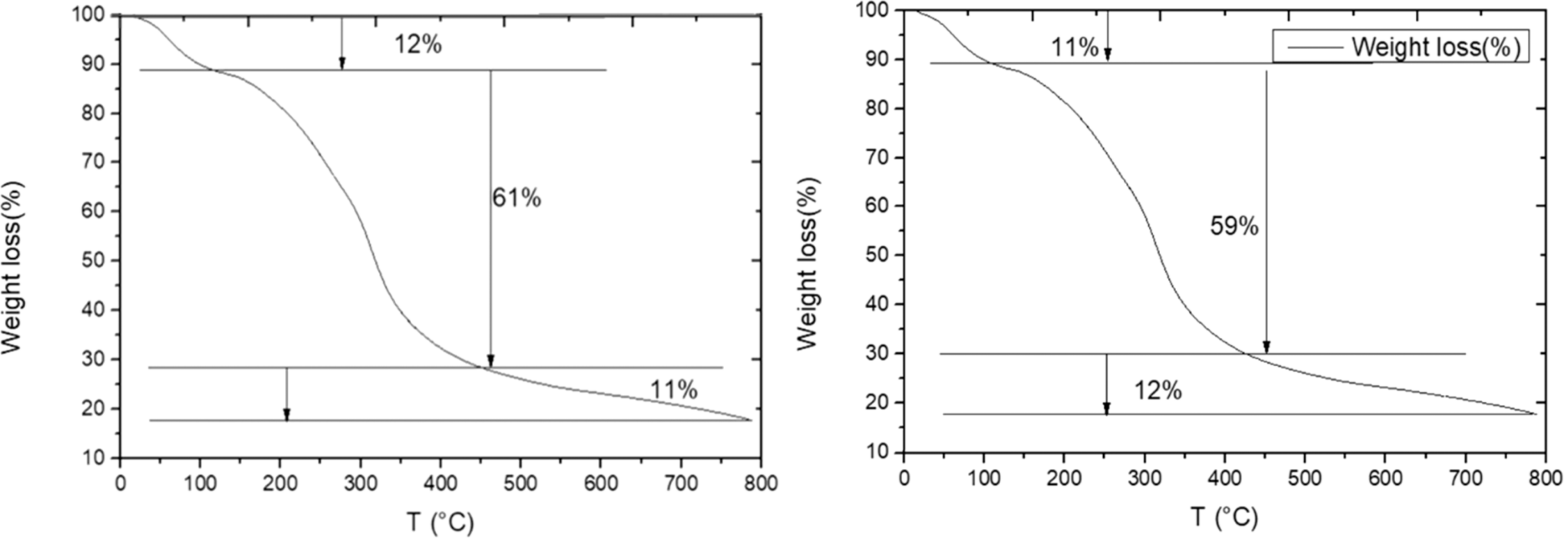
Thermogravimetric analysis of fish gelatin film with 0% of CDs (left) and 5% of CDs (right).

Thermogravimetric analysis was used to evaluate the thermal stability of the materials and the results are shown in Figure 3. A three-step weight loss was observed for all the samples. The first weight change occurred at 45–110 °C due to loss of water. The onset of the second step was at 125 °C and ended around 325 °C, and it was attributed to the breakdown of glycerol and of the gelatin chains. The last thermal degradation step started around 350 °C and it is consistent with the decomposition of gelatin. The addition of CDs caused no significant variation in the thermal stability of the materials.^59^

### Transmission Electron Microscopy (TEM) and Scanning Electron Microscopy (SEM)

The size of the CDs and their dispersion inside the gelatin matrix were investigated by transmission electron microscopy (TEM).

The carbon nanoparticles obtained from fish scales have near-spherical shape and a diameter of 10 nm, as already shown in our previous work^39^ (Figures S5–S7).

The micrographs of the films indicate that the CDs tend to aggregate resulting in relatively large clusters. With just 1% loading of nanoparticles, it is already possible to observe agglomerates with diameters in the order of 40 nm (Figures S11–S13). Increasing the loading of CDs, the clusters became bigger (Figures S14–S19) leading to a change in mechanical properties and in the decrease in optical transparency of the material (see Film Thickness and Mechanical Properties and Optical Properties). The pristine film, however, showed darker spots as well (Figures S8–S10) attributed to air bubbles due to the casting technique.

In addition, scanning electron microscopy (SEM) on the pristine film (15% glycerol, 0% CDs) was performed to observe the structure of the gelatin in the matrix. A dense and filamentous like structure was highlighted due to the organization of the renaturated gelatin (Figures S20–S25).

## 4 Conclusions

In this work, new hybrid completely biobased gelatin–CDs films with UV-shielding ability starting from fishery waste are described. Gelatin was extracted from mullet scales using a chemical protocol yielding type I collagen. The denaturation temperature was found to be lower than that of common mammalian gelatin making extraction possible under milder conditions. The carbon dots used to dope the films were obtained as 10 nm nanoparticles using a hydrothermal treatment starting from bass scales as a natural carbon and nitrogen source.^39^ The films were prepared by mixing fish gelatin, glycerol (15%), and CDs in different percentages (0, 1, 3, and 5%) by the casting technique. Here, 40 μm thick materials were obtained and improved mechanical properties were observed upon addition of the CDs: the EAB% increased from 27% (nonloaded film) to 40% (5% CDs) showing a clear plasticizing effect while, on the other hand, the stiffness decreased, probably due to the aggregation of the nanoparticles in the gelatin matrix. The films exhibited high water-solubility and decreasing WVP upon addition of the nanoadditive, indicating that the hybrid materials are less permeable to water.

From the optical point of view, the addition of CDs has only a limited effect on transparency (88.6% for the nonloaded vs 84.4% for 5% CDs) and on opacity (1.32 for nonloaded vs 1.61 for 5% CDs); while the material loaded with 5% CDs blocked almost the 70% of the UV radiation.

These results can pave the way toward the production of innovative films from waste with a view on the circular economy.

## Supporting Information Available

Viscoelastic properties of fish gelatin (Figures S1 and S2), absorbance UV–vis spectrum of gelatin/CDs films (Figure S3), differential scanning microscopy of gelatin/CDs films (Figure S4), transmission electron microscopy of gelatin/CDs films (Figure S5–S19), and scanning electron microscopy of gelatin/CDs films (Figure S20–S25) (PDF)
