# Supplementary material for: Fish-Waste-Derived Gelatin and Carbon Dots for Biobased UV-Blocking Films
Source: ACS Appl Mater Interfaces. 2022 Jul 25;14(30):35148–56. doi: 10.1021/acsami.2c11749 (PMC9354012; doi:10.1021/acsami.2c11749)
Supplement: Supplementary file 1 — am2c11749_si_001.pdf [file am2c11749_si_001.pdf]

SUPPORTING INFORMATION

Fish waste derived gelatin and carbon dots for biobased UV-blocking films

Carlotta Campalani[1], Valerio Causin[2], Maurizio Selva[1*,] Alvise Perosa[1*],

[1] Department of Molecular Sciences and Nanosystems, Università Ca' Foscari di Venezia, Via Torino 155, 30172 Venezia Mestre, Italy; carlotta.campalani@unive.it (C.C.); alvise@unive.it (A.P.); selva@unive.it (M.S.)

[2] Dipartimento di Scienze Chimiche, Università di Padova, via Marzolo 1, 35131 Padova, Italy; valerio.causin@unipd.it (V.C.)

*Correspondence:

Table of Content

1. Viscoelastic properties of fish gelatin: Rheological profiles
2. UV-Vis absorbance spectrum of gelatin-CDs films
3. Differential Scanning Calorimetry of gelatin-CDs films
4. Transmission Electron Microscopy of gelatin-CDs films
5. Scanning Electron Microscopy of gelatin-CDs films

1. VISCOELASTIC PROPERTIES OF FISH GELATIN: RHEOLOGICAL PROFILES

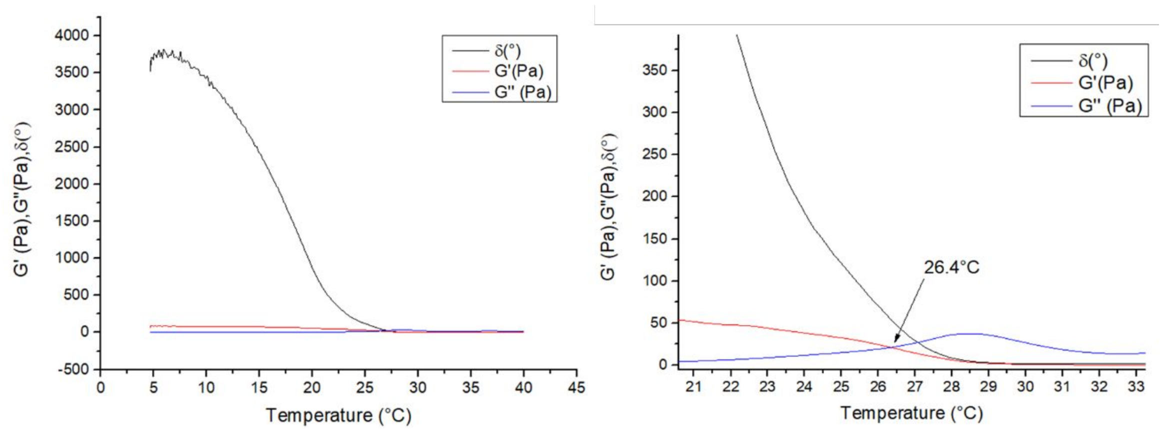

Figure S1. Rheological profile from -5 to 40 °C, at 1 Hz (heating ramp: 5 °C/min). Left: full profile; right: zoom on the cross-over point

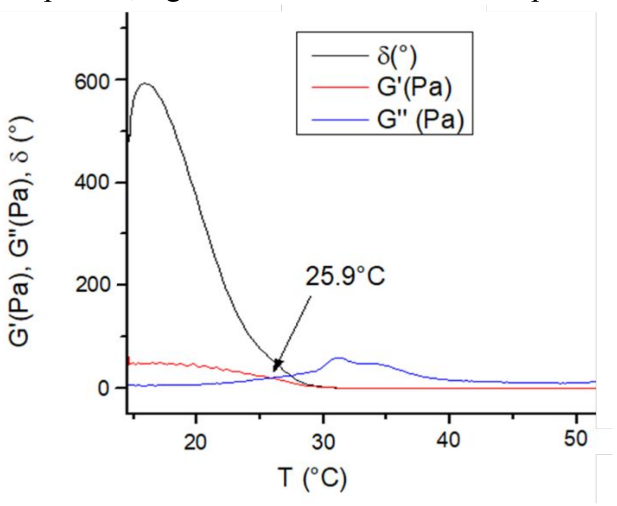

Figure S2. Rheological profile from 12 to 90 °C, at 0.3 Hz (heating ramp: 5 °C/min)

2. UV-Vis ABSORBANCE SPECTRUM OF GELATIN-CDs FILMS

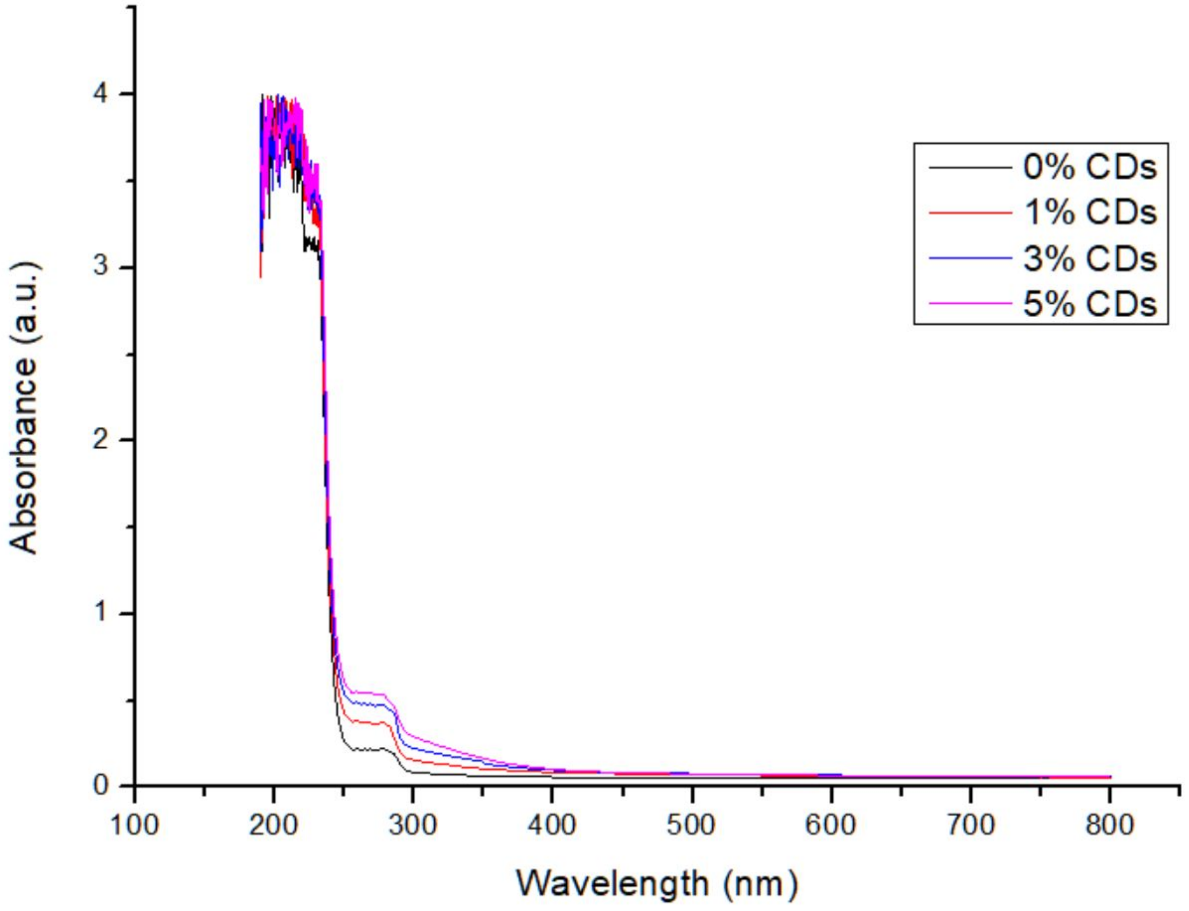

Figure S3. Uv-Visible absorbance spectrum of gelatin films with different concentrations of CDs (0% black line, 1% red line, 3% blue line and 5% pink line).

3. DIFFERENTIAL SCANNING CALORIMETRY OF GELATIN-CDs FILMS

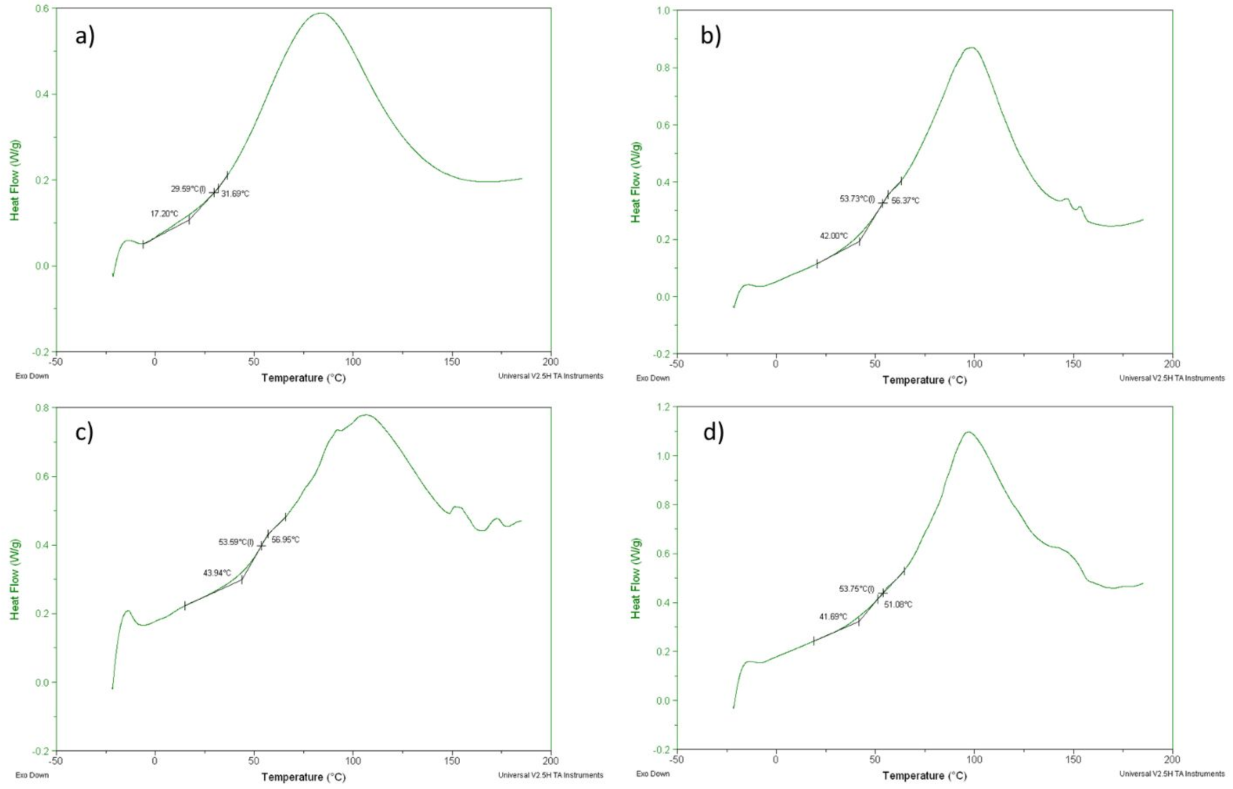

Figure S4. DSC curves of fish gelatin films containing (a) 0% of CDs, (b) 1% of CDs, (c) 3% of CDs and (d) 5% of CDs.

4. TRANSMISSION ELECTRON MICROSCOPY OF GELATIN-CDs FILMS

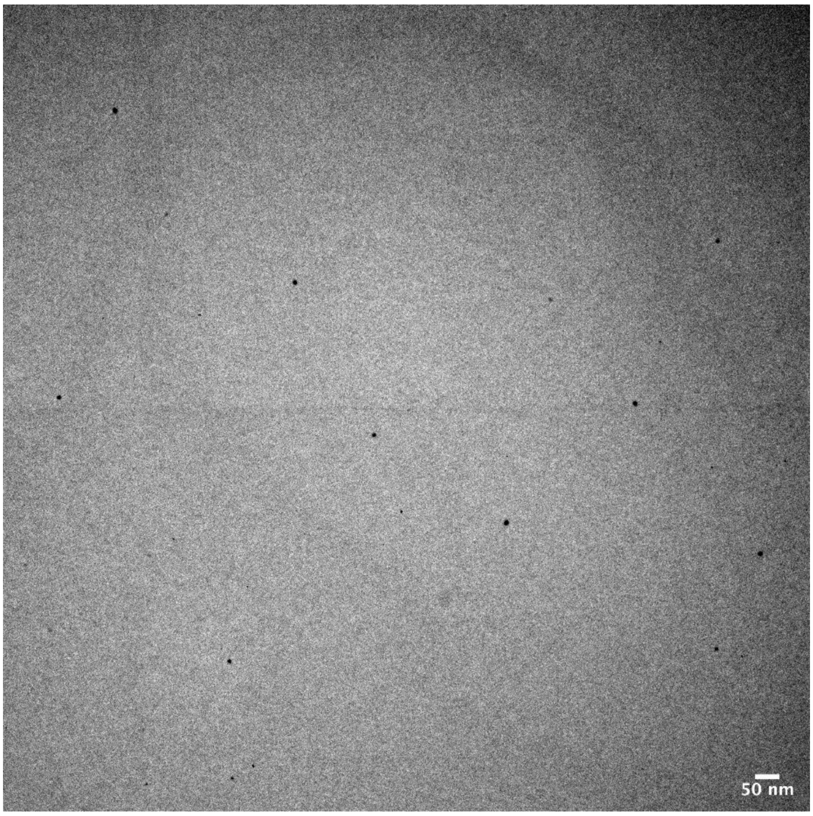

Figure S5. TEM micrographs of fish derived CDs.

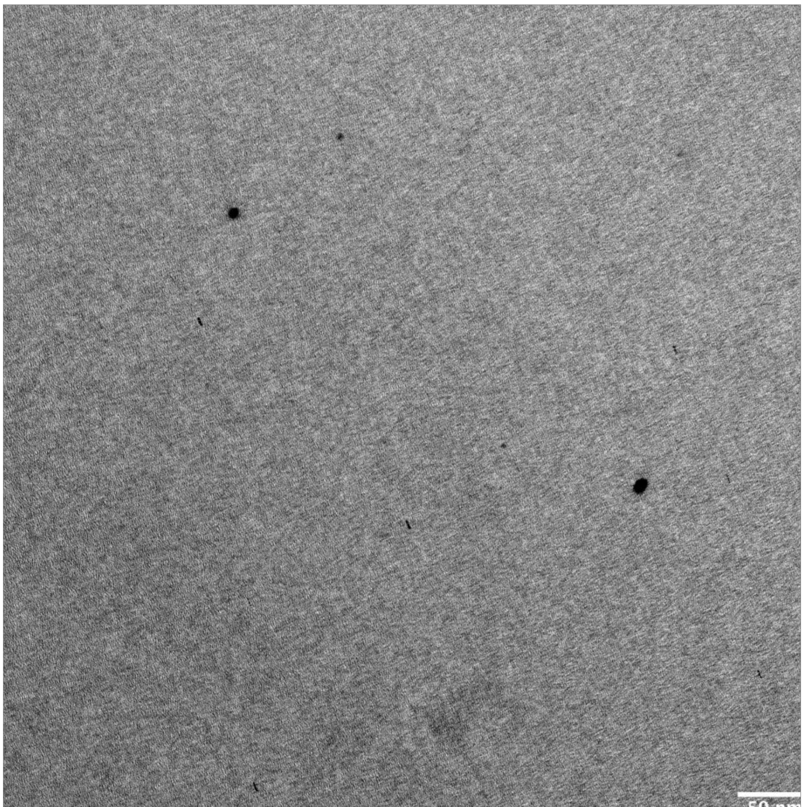

Figure S6. TEM micrographs of fish derived CDs.

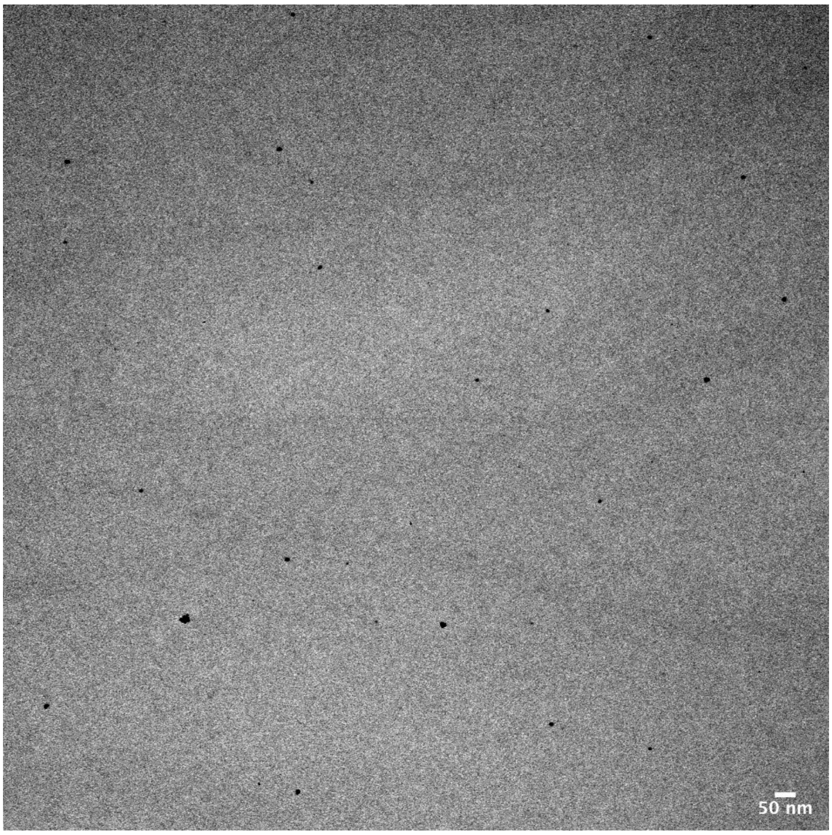

Figure S7. TEM micrographs of fish derived CDs.

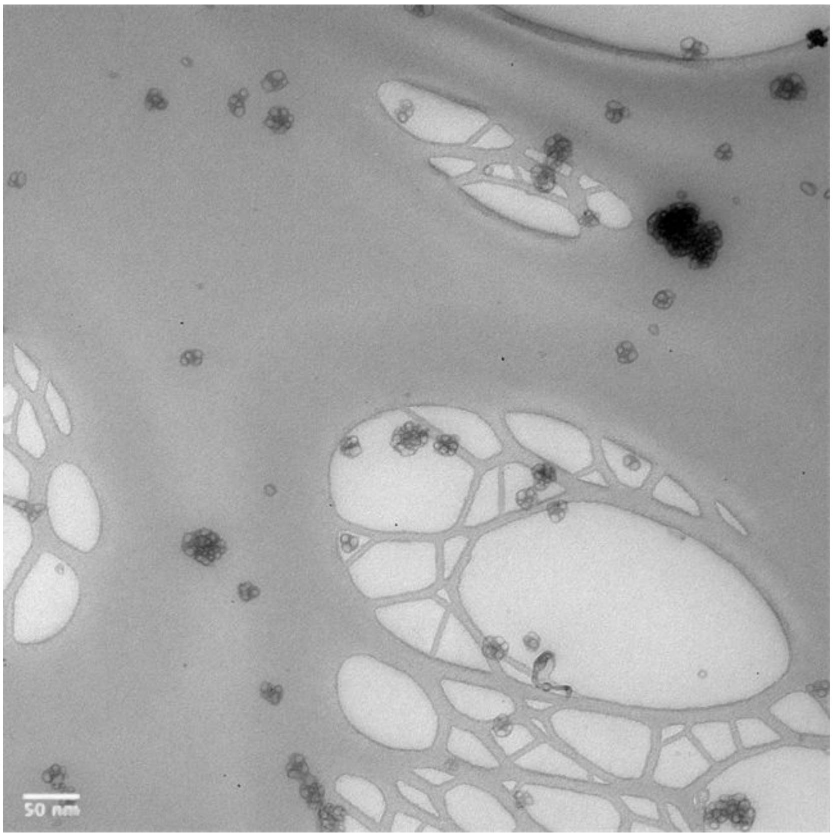

Figure S8. TEM micrographs of gelatin-CDs films with 0% of CDs.

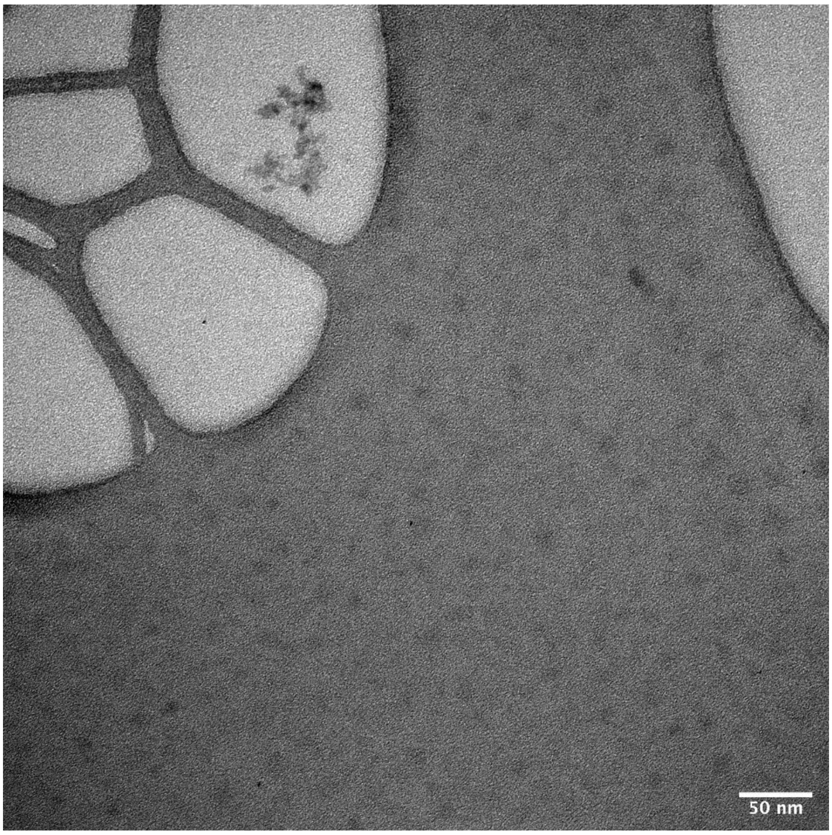

Figure S9. TEM micrographs of gelatin-CDs films with 0% of CDs.

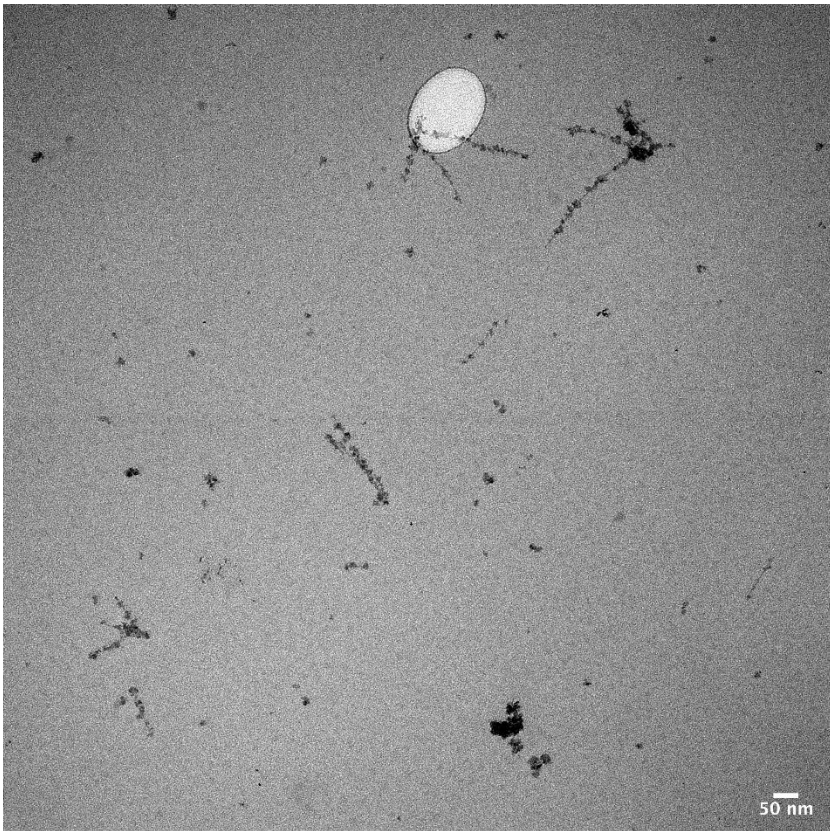

Figure S10. TEM micrographs of gelatin-CDs films with 0% of CDs.

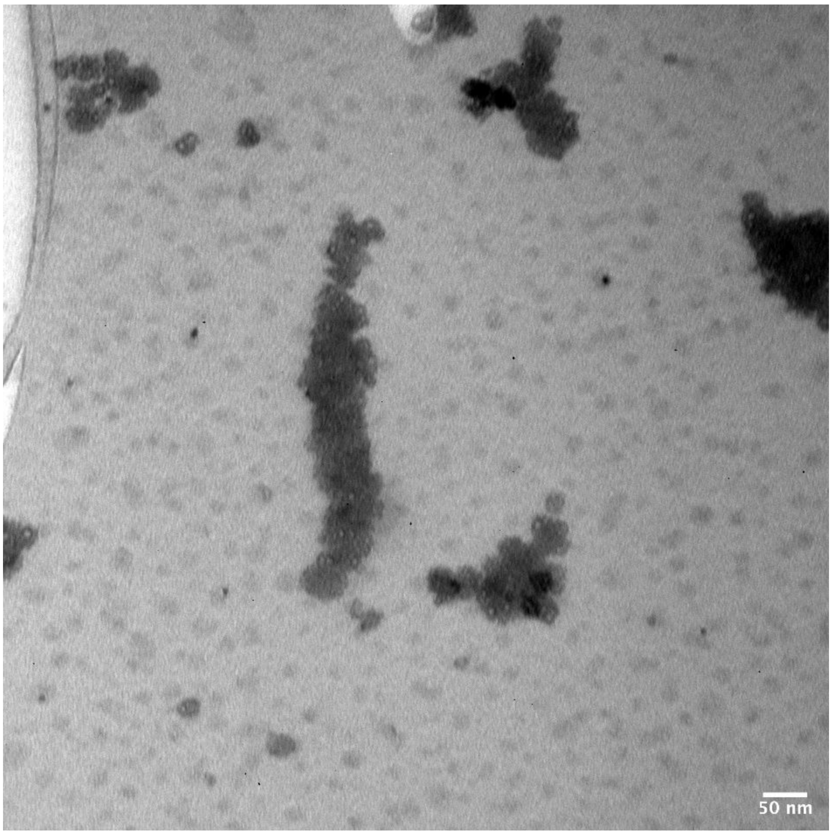

Figure S11. TEM micrographs of gelatin-CDs films with 1% of CDs

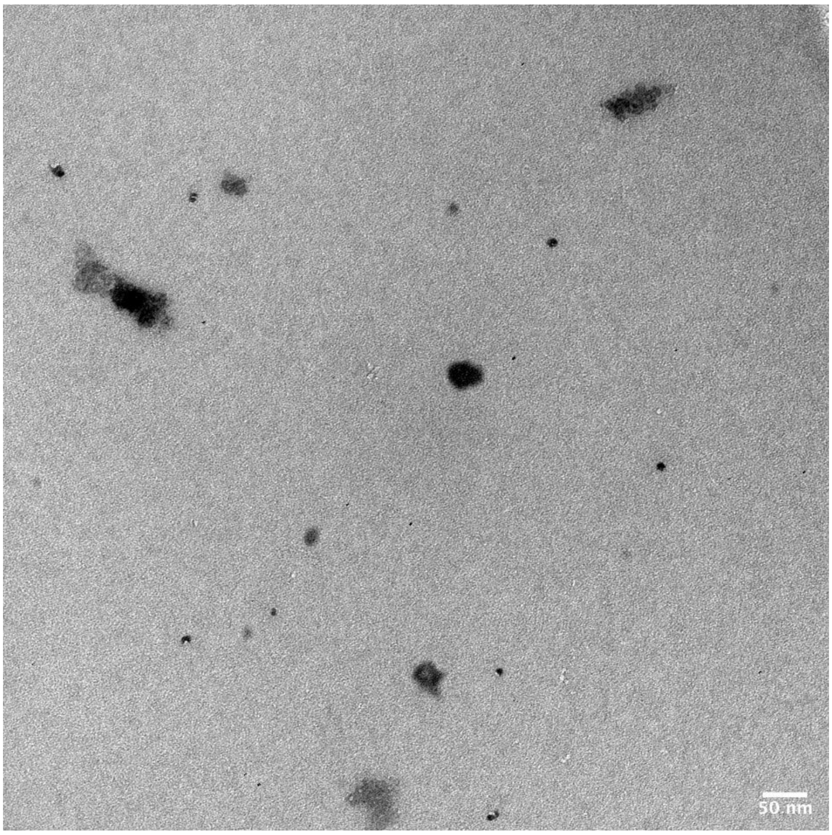

Figure S12. TEM micrographs of gelatin-CDs films with 1% of CDs

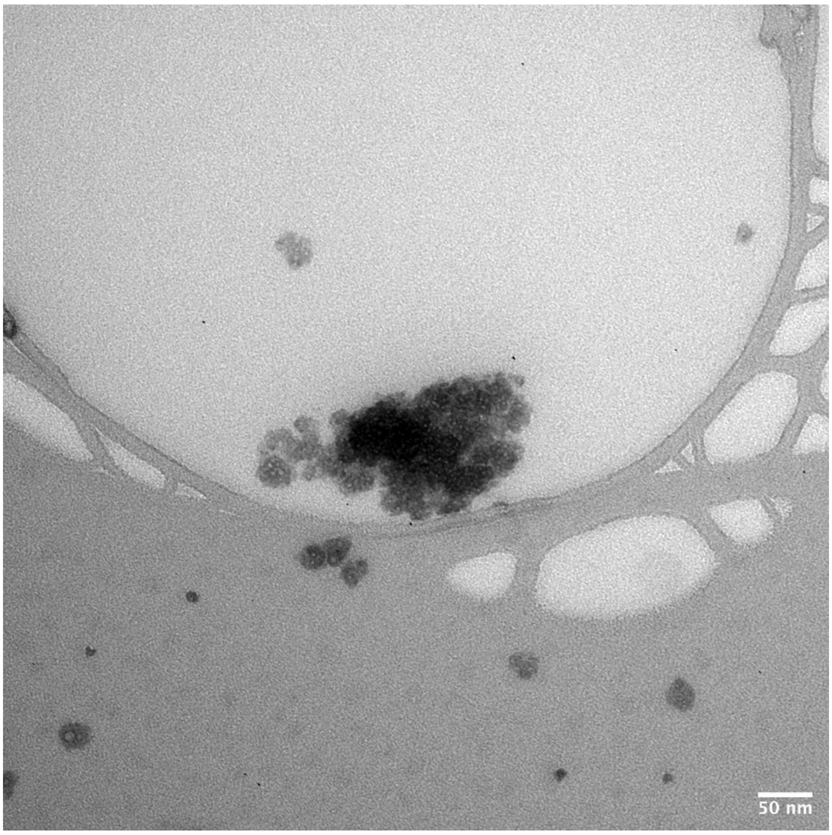

Figure S13. TEM micrographs of gelatin-CDs films with 1% of CDs.

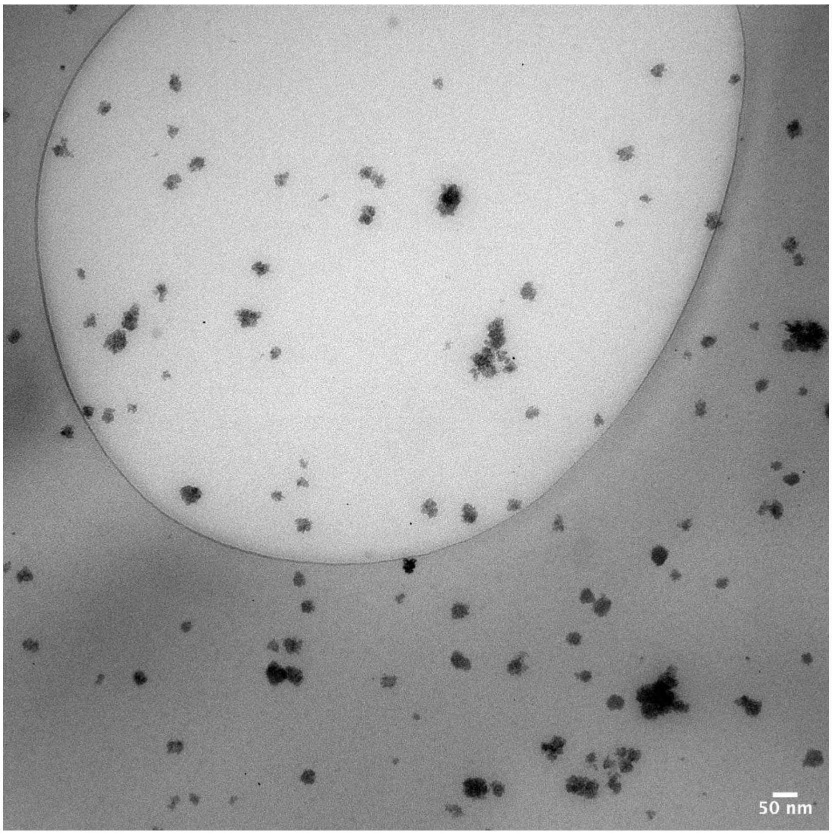

Figure S14. TEM micrographs of gelatin-CDs films with 3% of CDs.

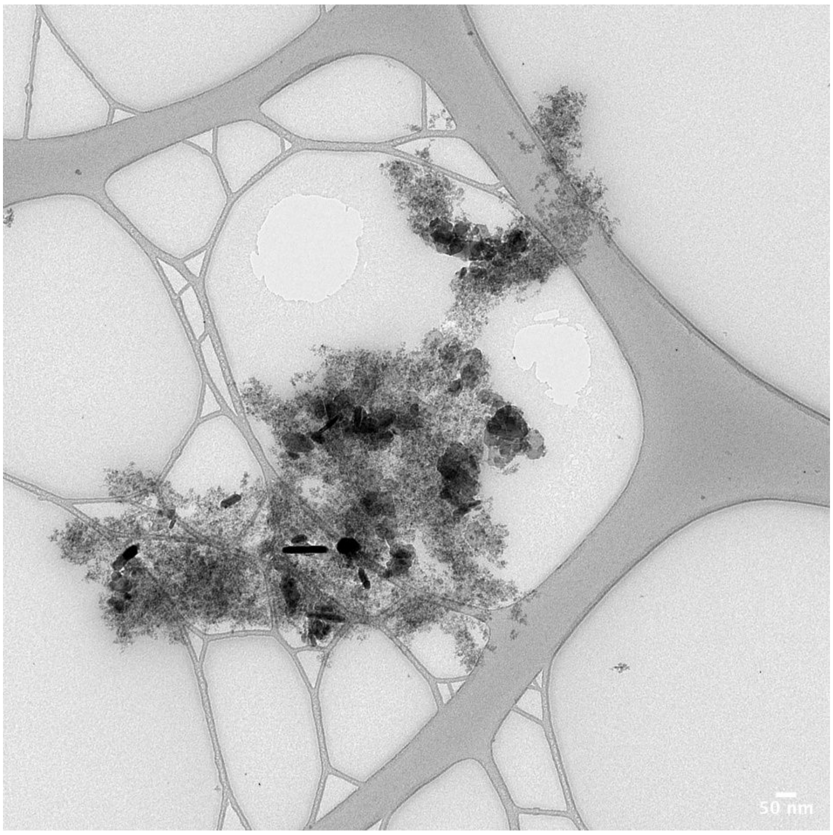

Figure S15. TEM micrographs of gelatin-CDs films with 3% of CDs.

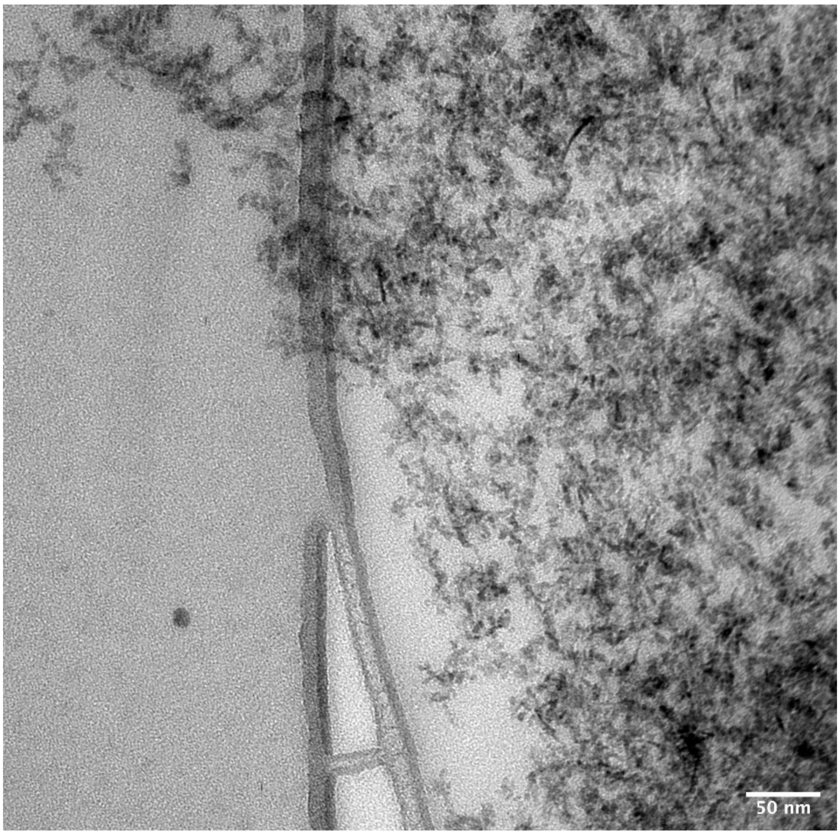

Figure S16. TEM micrographs of gelatin-CDs films with 3% of CDs.

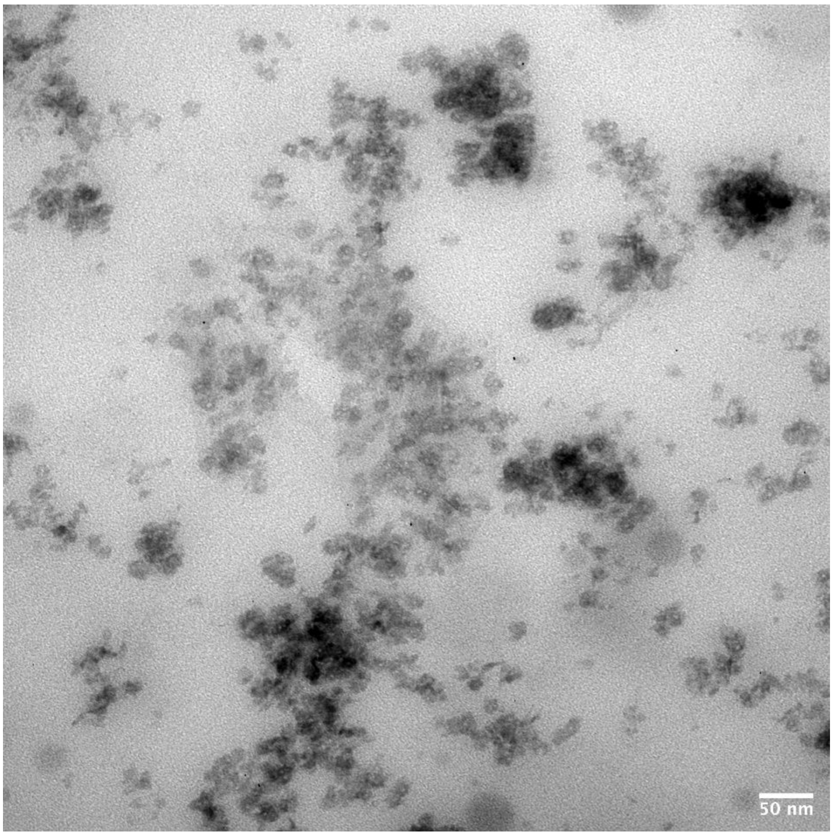

Figure S17. TEM micrographs of gelatin-CDs films with 5% of CDs.

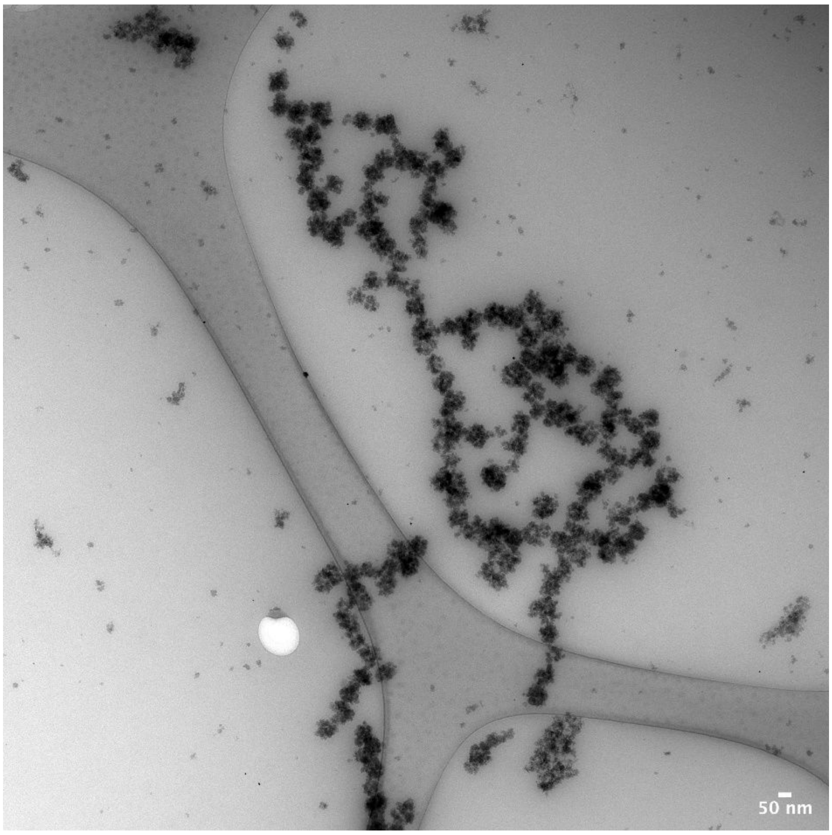

Figure S18. TEM micrographs of gelatin-CDs films with 5% of CDs.

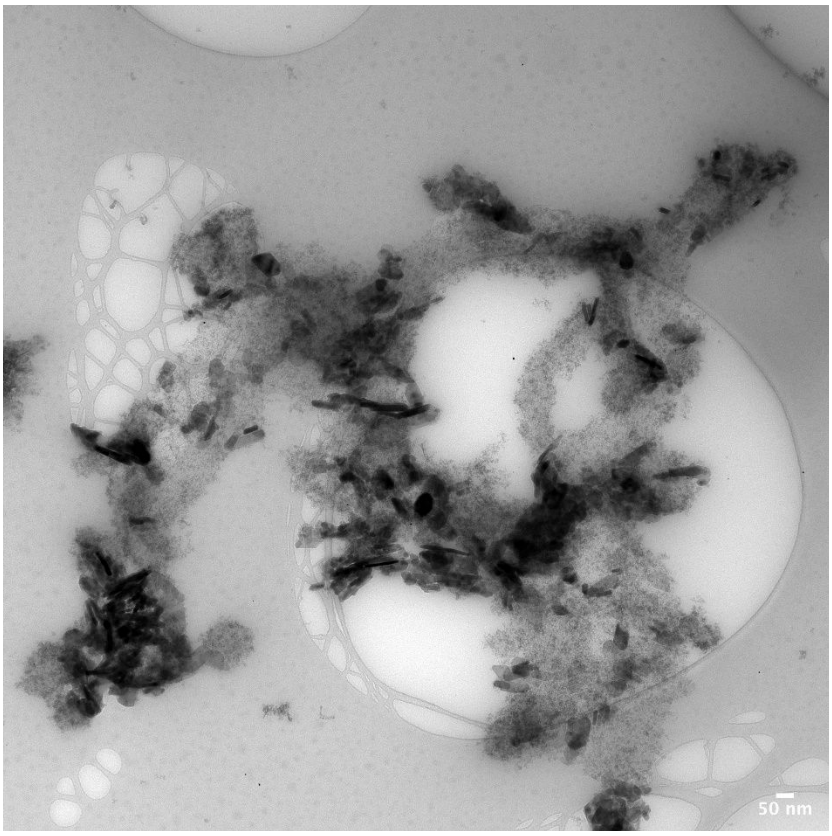

Figure S19. TEM micrographs of gelatin-CDs films with 5% of CDs.

5. SCANNING ELECTRON MICROSCOPY OF GELATIN-CDs FILMS

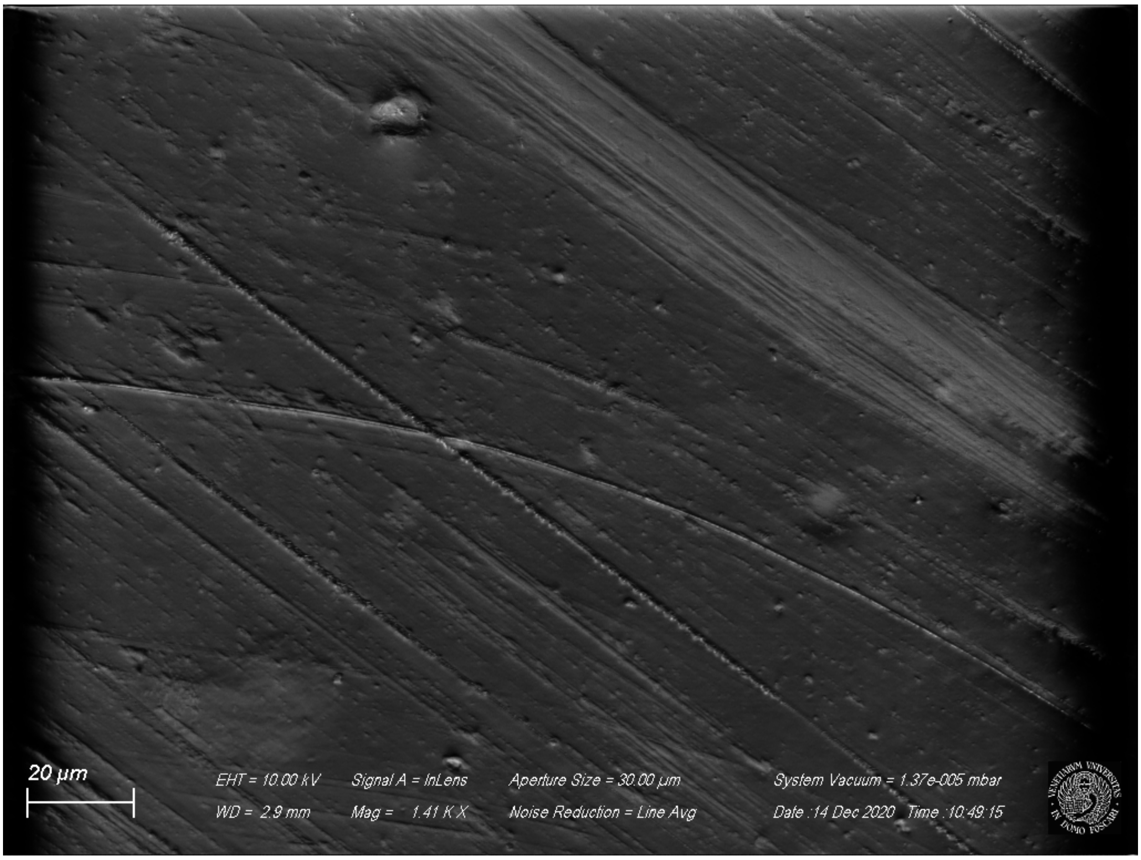

Figure S20. SEM micrograph (X 1.41K) of the pristine fish gelatin films.

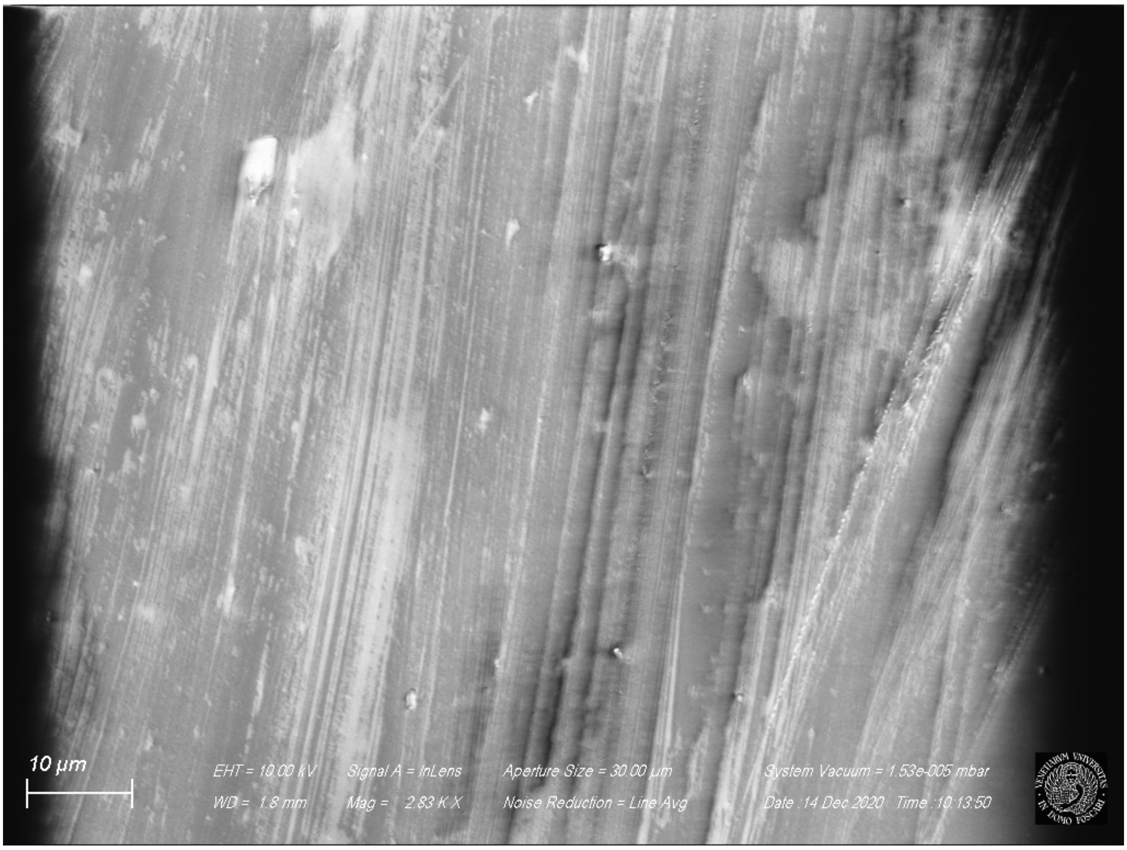

Figure S21. SEM micrograph (X 2.83K) of the pristine fish gelatin films.

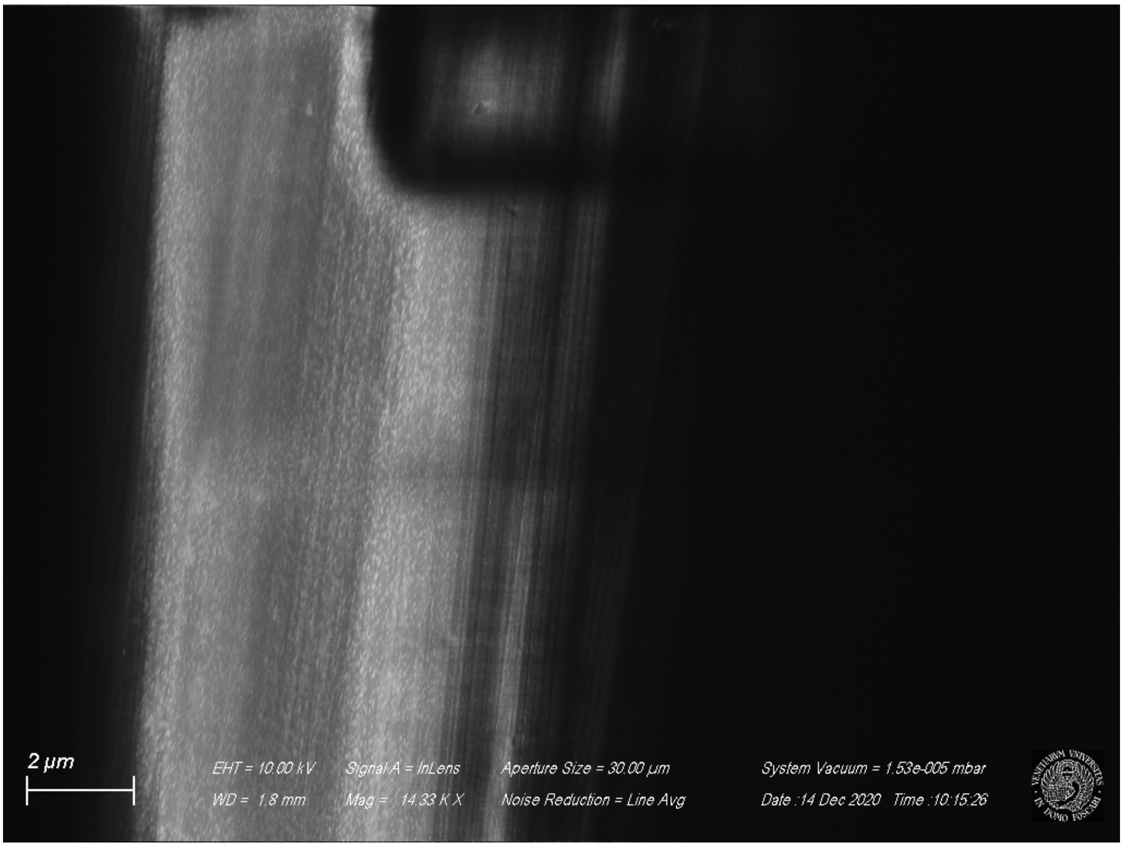

Figure S22. SEM micrograph (X 14.33K) of the pristine fish gelatin films.

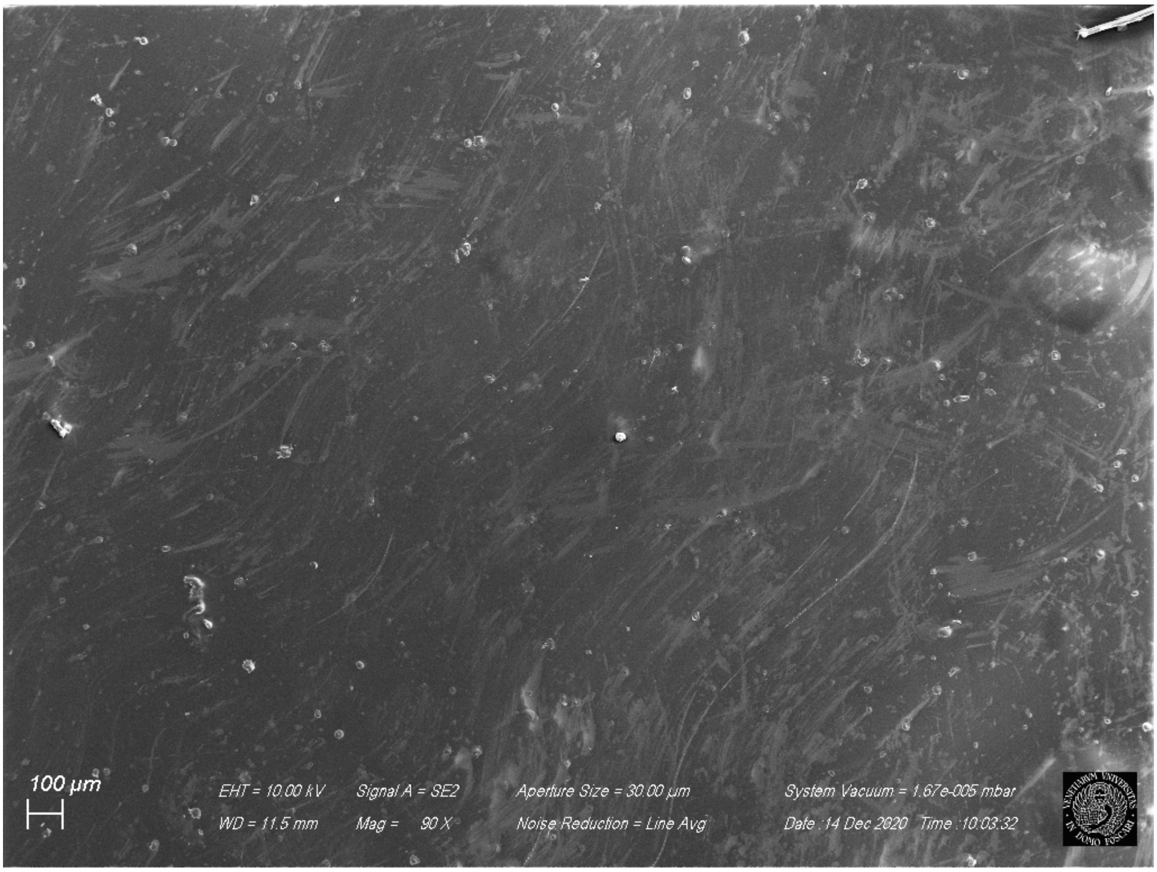

Figure S23. SEM micrograph (X 90) of the pristine fish gelatin films.

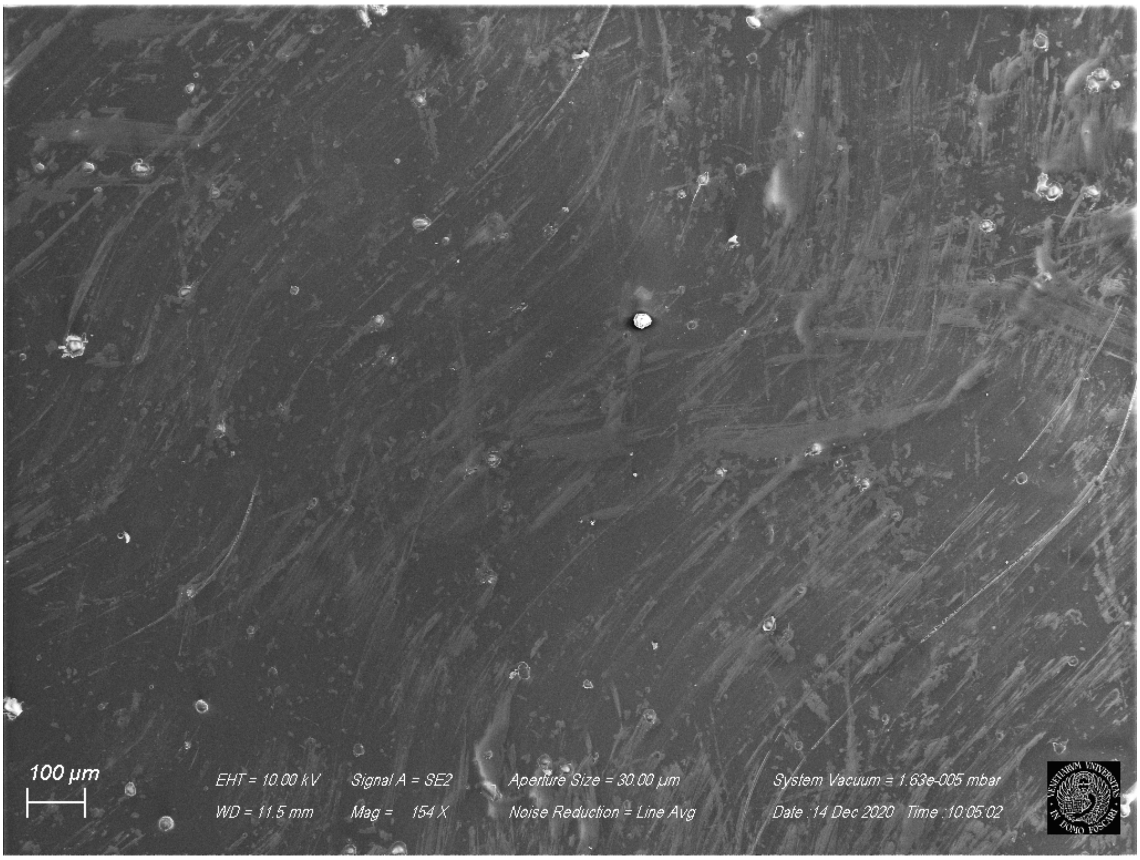

Figure S24. SEM micrograph (X 154) of the pristine fish gelatin films.

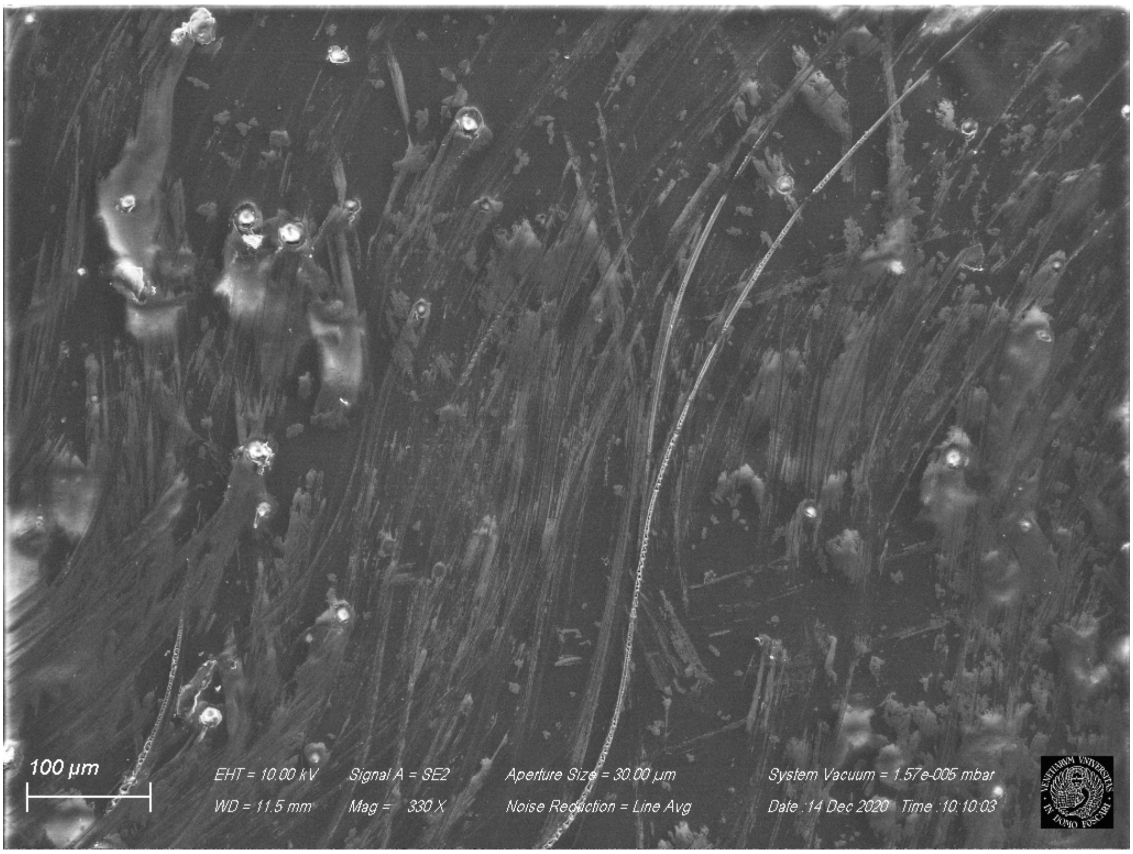

Figure S25. SEM micrograph (X 330) of the pristine fish gelatin films.
